# Metabolic profiling of historical and modern wheat cultivars using proton nuclear magnetic resonance spectroscopy

**DOI:** 10.1038/s41598-021-82616-3

**Published:** 2021-02-04

**Authors:** Rachana Poudel, Fatema Bhinderwala, Martha Morton, Robert Powers, Devin J. Rose

**Affiliations:** 1 Department of Food Science and Technology, University of Nebraska-Lincoln, 1901 North 21st Street, Lincoln, NE 68588-6205 USA; 2 Department of Chemistry, University of Nebraska-Lincoln, Lincoln, NE 68588-0304 USA; 3 Nebraska Center for Integrated Biomolecular Communication, University of Nebraska-Lincoln, Lincoln, NE 68588-0304 USA; 4 Department of Agronomy and Horticulture, University of Nebraska-Lincoln, Lincoln, NE USA

**Keywords:** Natural variation in plants, NMR spectroscopy

## Abstract

To determine changes in the grain components between historical and modern wheat (*Triticum aestivum* L.) cultivars, wholemeal flours from 19 wheat cultivars and 2 landraces released or introduced between 1870 and 2013 and grown over two crop years were extracted using hydroalcoholic solution and analyzed using one dimensional ^1^H NMR spectral profiling. Grain yield, grain volume weight (GVW), and grain protein concentration were also measured. Grain yield increased while protein concentration decreased by release year (p < 0.001). Increasing trends (p < 0.01) were observed for tryptophan, sum of the measured amino acids, chlorogenic acid, ferulic acid, vanillic acid, and sum of the measured phenolic acids. Grain yield, phenolic acids, and tryptophan were mainly associated with modern cultivars, whereas grain protein concentration and GVW were associated with historical cultivars. The findings from this study showed changes in concentration of grain components over a century of breeding that may have implications for grain quality and human health.

## Introduction

The association of humans with wheat cultivation and consumption began about 10,000 years BC^1^. Early wheat farmers selected wheats based on desirable characteristics, but without any defined methodological approaches or knowledge of genetics or nutritional composition of grains. Only in the last century have modern wheat breeding programs been initiated to develop semi-dwarf and high yielding varieties of wheat with improved end-use quality^2^. Since then, the selective breeding of wheat has contributed to the development of many modern wheat cultivars with improved yield and agronomic characteristics^2^. Furthermore, many recent studies have been focused on improving genetic diversity in wheat germplasm for the development of cultivars with better end-use properties^3^. Remarkably, these gains have been achieved in the presence of emerging biotic and abiotic stresses and dramatic climate change^4^.

Despite the advancements in wheat production and quality, wheat breeders and wheat breeding programs have faced criticism from the general public. Critics have expressed concerns that modern wheat may contain new components that have adverse impacts on human health^5^. Although such concerns have not been supported by scientific investigation^5,6^, comparative studies have been carried out to quantify the changes in compositional properties of wheat with respect to the year that the cultivars were introduced^7^. Accordingly, a decrease in grain protein and mineral concentrations (iron, phosphorus, sulfur, and zinc) were reported for modern wheat compared to historical cultivars^8,9^, This decrease in nutritional value has been attributed to the yield dilution phenomenon^10^.

Reports on the nutritional composition of historical and modern wheats have been contradictory among some studies. For example, total phenolic compounds concentration, number of phenolic compounds, and their isomers in historical wheats were reported to be higher than in modern wheats^11,12^, whereas contrasting results were reported in other studies^13^. Due to these contradictory findings further investigation is needed to answer if wheat breeding practices caused changes in the chemical composition which ultimately affect nutritional and functional properties of modern wheat compared to historical wheat cultivars.

The use of ^1^H NMR-based metabolomics approach to study the diversity of metabolites in cereals have been established^14^. Simple sample preparation techniques and detection of metabolites, regardless of chemical properties (e.g., molecular weight) present in the complex sample matrix, provided they have nuclei with a non-zero spin quantum number are few advantages of NMR based approaches. In this study, we used an NMR-based metabolomics approach to determine the changes in compositional properties of historical versus modern wheat cultivars adapted to the Great Plains region of the United States, a major hard winter wheat-producing region of the Unites. The hypothesis was that the concentrations of some metabolites would vary depending on release year of the cultivar. However, due to contradictory reports in the literature it was difficult to predict how the metabolites would vary. A logical assumption was that many metabolites would decrease with release year due to a yield dilution effect^9,10,13,15^.

## Results and discussion

### Plant materials

As a result of breeding efforts, great strides in agronomic properties and grain quality have been reported^2,16^. However, changes in minor grain components have either not been reported or show inconsistent results. Therefore, in this study we analyzed a set of two landraces and 19 wheat cultivars released over a century of wheat breeding in the Great Plains of the US (Table 1). The landraces and cultivars included in this study were selected because they represented the genetic diversity and production history of the Great Plains. These cultivars contributed to the pedigree of recent elite cultivars and occupied considerable acreage at their time. Moreover, this set of cultivars was selected due to their known adaptation to the location of the study.

**Table 1:** Release year, origin, and plant introduction (PI) or cereal introduction (CI) number. For the wheat cultivars used in this study.

Cultivar	Year of introduction, release, or PVP	Place of origin (US)	PI or CI number
Turkey	1870	Landrace	CItr 5757
Kharkof	1900	Landrace	PI 5641
Cheyenne	1933	UNL	CItr 8885
Red Chief	1940	Kansas	CItr 12,109
Wichita	1944	KSU	CItr 11,952
Warrior	1960	UNL	CItr 13,190
Lancer	1963	USDA/UNL	CItr 13,547
Triumph 64	1964	OKS	CItr 12,132
Sturdy	1966	TAM	CItr 13,684
Scout 66	1967	UNL	CItr 13,996
Clark’s Cream	1972	Kansas	PI 476,305
Centurk 78	1978	UNL	CItr 17,724
Centura	1983	UNL	PI 476,974
TAM 107	1984	TAM	PI 495,594
Wesley	1998	USDA/UNL	PI 605,742
Jagalene	2002	Monsanto	PI 631,376
Anton	2007	USDA	PI 651,044
Overland	2007	UNL	PI 647,959
Settler CL	2008	UNL	PI 653,833
Mattern	2012	USDA/UNL	PI 665,947
Freeman	2013	UNL	PI 667,038

*PVP* plant variety protection, *USDA* US Department of Agriculture, *UNL* University of Nebraska-Lincoln, *TAM* Texas A&M University, *OKS* Oklahoma State University, *KSU* Kansas State University; PI or CI obtained from the USDA-Agricultural Research Service National Plant Germplasm System Database: https://npgsweb.ars-grin.gov/gringlobal/search.aspx.

### Changes in economic and agronomic traits

**Figure 1:**
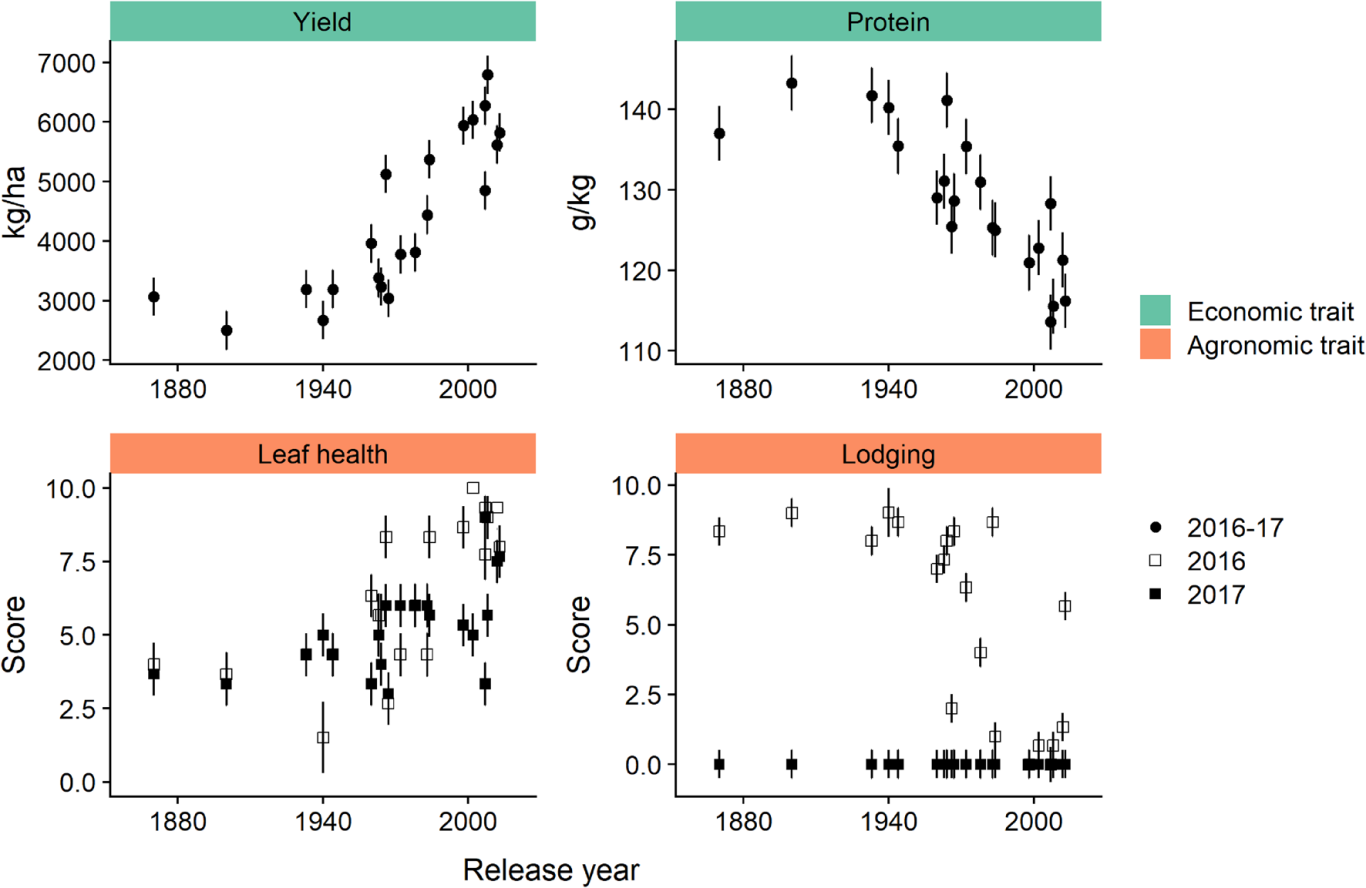
Scatter plots of cultivar year of introduction for economic and agronomic traits that showed significant changes by release year (as reported in Table 3). Where the harvest year × release year interaction was not significant, each point represents the least squares mean of a cultivar or landrace across two harvest years (2016–2017); where the harvest year × release year interaction was significant least squares mean of a cultivar or landrace are shown for 2016 and 2017 separately; leaf health and lodging were scored on a scale of 0–10 where 0 = no green on flag leaf and 10 = flag leaf completely green for leaf health and 0 = no lodging and 10 = completely flat for lodging; error bars show standard error; plot generated using the ‘ggplot2’ and ‘cowplot’ packages in R^38,40,41^.

Grain yield and protein concentration varied significantly depending on release year (Table 2). Grain yield increased at the rate of 28 kg/ha/year; whereas, grain protein concentration decreased at the rate of 0.2 g/kg/year (Fig. 1). Guttieri et al.^9^ showed similar results when grain yield and grain protein concentration were analyzed according to release year of historic and modern wheat cultivars. The simultaneous increase in yield and decrease in protein concentration were attributed to the yield dilution effect^9,10,15^. The broad-sense heritability of grain yield (*H*^2^ = 0.94) was higher than previous studies, which was associated with high genetic variance (cultivars) and low variance due to the non-significant cultivar × harvest year interaction (Table 2). The heritability of grain volume weight (*H*^2^ = 0.63), and protein concentration (*H*^2^ = 0.87) were in line with previous studies^9,10,15^. Leaf health and lodging showed significant release year and harvest year interactions (Table 2). The interaction in lodging was because there was only lodging observed in the 2016 harvest year. The leaf health and lodging results showed improved health and lower lodging problems with respect to the increasing release year (Fig. 1). These results were in line with previous reports showing improvements in agronomic traits in modern versus historical wheat cultivars^16,17^.

**Table 2:** Analysis of variance (mean squares) and broad sense heritability of economic and agronomic traits and metabolites in whole wheat flour from modern and historical wheat varieties.

Variable	Units	Release year (RY)	Harvest year (HY)	Rep(HY)	RY × HY	Residuals	Heritability
**Economic traits**							
Grain yield	kg/ha	135,200,000***	36,660	1,732,000	25,800	1,103,000	0.94
Grain volume weight	kg/hL	2.4	11.1	2.3	14.2	2.2	0.63
Protein	%	59.9***	0.9	4.9	0.7	0.7	0.87
**Agronomic traits**							
Leaf health		200***	16*	0.6	18*	3	0.69
Lodging		188***	202***	1.1	188***	3.6	0.01
**Amino acids**							
Alanine	mg/kg	1630	998	2280	1946	1505	0.31
Asparagine	mg/kg	4160	262,400	111,400	287,200**	37,380	0.41
Betaine	mg/kg	669	692	1646	340	7500	0.16
Isoleucine	mg/kg	1263	75	2847	133	1232	0.78
Leucine	mg/kg	143	9167	4236	10,000	2574	0.24
Threonine	mg/kg	13,330	500	2810	69	5489	0.34
Tryptophan	mg/kg	1,544,000***	223,900	175,800	265,200	48,840	0.78
Valine	mg/kg	18	4057	1425	4716	1566	0.61
Sum amino acids	mg/kg	1,749,000**	1,940,000	470,400	2,167,000**	257,000	0.59
**Carbohydrates**							
Glucose	mg/kg	55,650,000**	42,480,000	10,040,000	46,960,000	8,469,000	0.34
Maltose	mg/kg	376,200	1,420,000	3,337,000	1,670,000	2,573,000	0.49
Sucrose	mg/kg	315,200,000***	3,989,000	13,930,000	11,150,000	23,820,000	0.80
Sum sugars	mg/kg	562,200,000**	6,655,000	9,990,000	2,346,000	60,550,000	0.70
**Phenolic acids**							
Chlorogenate	mg/kg	1,418,000***	107,800	168,700	146,800	65,340	0.67
Ferulate	mg/kg	230,900***	842	92,570	2425	13,660	0.69
Syringate	mg/kg	9313	2698	4976	2168	1718	0.68
Vanillate	mg/kg	267,400***	38,940	44,830	46,530	8700	0.58
Sum phenolics	mg/kg	4,854,000***	365,500	988,100	501,300	204,600	0.70
**Carboxylic acid**							
Fumarate	mg/kg	8119***	64	833	2681**	854	0.70
df		1	1	4	1	115	

*df* degrees of freedom.

*P < 0.05; **P < 0.01; ***P < 0.001.

### Changes in wheat metabolite concentrations

**Figure 2:**
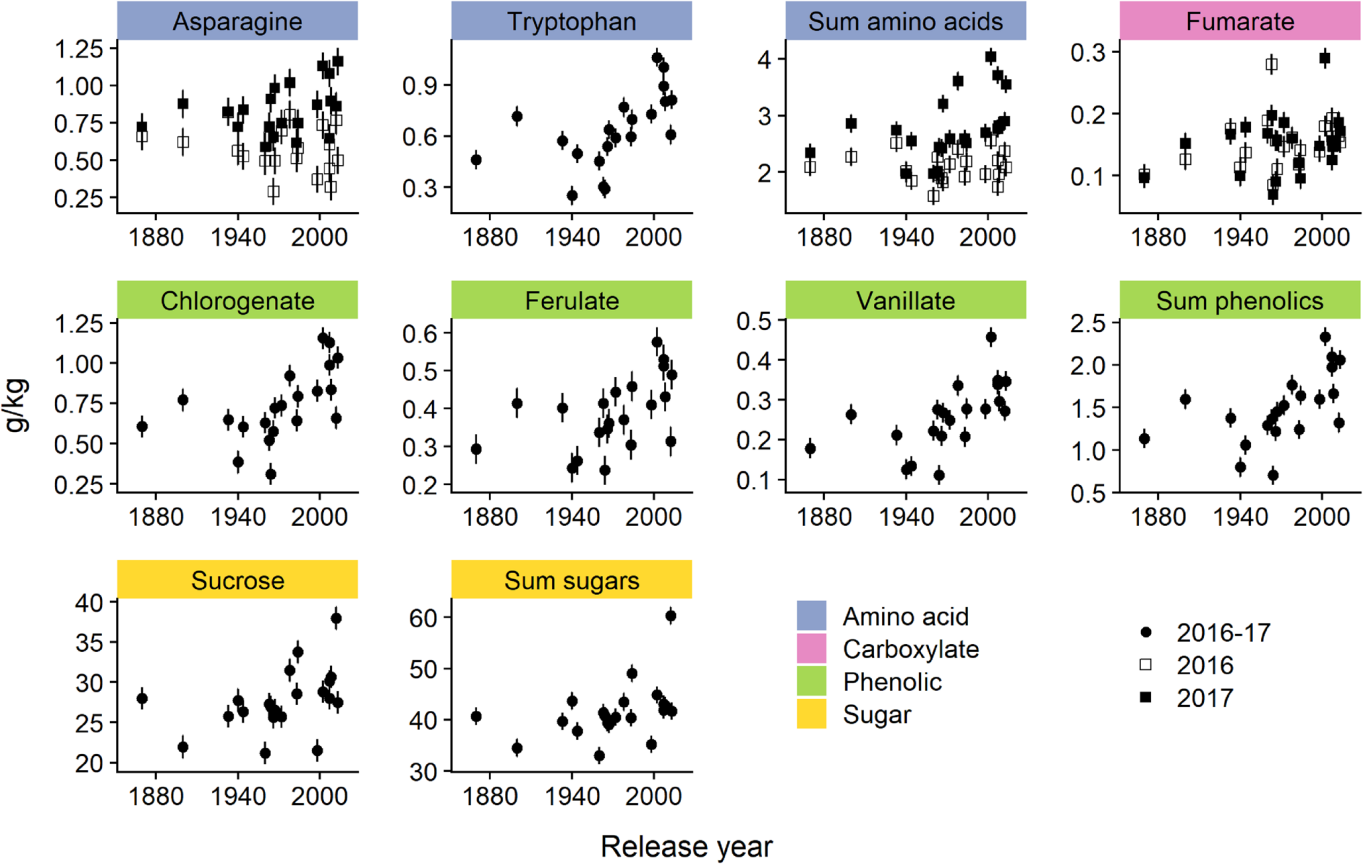
Scatter plots of cultivar year of introduction for metabolites that showed significant changes by release year (as reported in Table 3). Where the harvest year × release year interaction was not significant, each point represents the least squares mean of a cultivar or landrace across two harvest years (2016–2017); where the harvest year × release year interaction was significant least squares mean of a cultivar or landrace are shown for 2016 and 2017 separately; error bars show standard error; plot generated using the ‘ggplot2’ and ‘cowplot’ packages in R^38,40,41^.

Free asparagine and the sum of the measured free amino acids varied significantly by the interaction between release year and harvest year (Table 2). Specifically, asparagine and the sum of the measured amino acids showed dramatically different effects by release year; in 2017 there was a significant increase in these compounds across release years while in 2016 there was no trend across release year (Fig. 2). The mean asparagine concentration varied between 0.47 and 0.93 g/kg among wheat cultivars, which falls in the range as reported in 150 bread wheat lines^18^. A decrease in asparagine concentration between historical and modern wheats was reported in these 150 bread wheat lines grown over 1 year^18^. Our results were similar to these reports in 2016 but not in 2017, suggesting a differential environmental impact on the concentration of these compounds.

The mean free tryptophan concentration varied between 0.25 and 1.07 g/kg among wheat cultivars which was lower than those reported in 24 Australian wheats (mean tryptophan = 1.56 g/kg)^19^. This difference may be due one or combination of variation due genotype, growing environment, agronomic practices, and analytical approaches. The tryptophan concentration increased at a rate of 4.5 mg/kg/year wheat (Table 2; Fig. 2). The increase in tryptophan in modern cultivars is interesting because tryptophan was identified as a major bitter compound in the sensory analysis of whole wheat bread^20^. Furthermore, a sensory evaluation of bread made from historical and modern cultivars showed a preference for historical wheat bread^21^. Although the taste of a final product is affected by multiple factors (e.g., chemical composition, formulation, and production process), it would be interesting to identify if the sensory perception of bread and other products are related to the release year.

The mean glucose (3.41–7.46 g/kg), maltose (6.21–10.9 g/kg), and sucrose (21.7–38.4 g/kg) concentrations were comparable to the concentrations reported in previous studies^22^. Glucose, sucrose, and the sum of the measured sugars varied significantly by release year (Table 2). The significant effect of release year on glucose concentration was due to an outlier (all replicates of Mattern wheat had an abnormally high concentration in 2016). Therefore, ANOVA was performed removing the outlier, and, as expected, the relationship between glucose concentration and release year was no longer significant (p = 0.15). However, sucrose and the sum of the measured sugars increased at a rate of 0.052, and 0.054 g/kg/year, respectively (Fig. 2). The increase in total carbohydrates (sweetness) and tryptophan concentration (bitterness) in modern wheat hinted that the taste of final products may not be different as both flavors are increasing and may mask each other’s effect.

The range of mean concentrations of ferulic acid (0.22–0.58 g/kg), vanillic acid (0.11–0.46 g/kg), and syringic acid (0.059–0.19 g/kg) were comparable to the concentrations reported in previous studies^23^. Chlorogenate, ferulate, vanillate, and the sum of the measured phenolic acids varied significantly by release year (Table 2). Chlorogenate (4 mg/kg/year), ferulate (1.4 mg/kg/year), vanillate (1.9 mg/kg/year), and the sum of the phenolics (7.4 mg/kg/year) showed increasing trends with respect to release year (Fig. 2). Similar to the observations reported herein, higher phenolic concentrations were observed in modern wheat cultivars compared to old cultivars^13,24^. Conversely, higher polyphenol concentrations were previously reported in old wheat cultivars^12^. These contrary results may be attributed to differences in genetics, environment or quantification methods. The increase in phenolic acids may improve the stability of flour and increase the nutritional properties; however, these phenolic acids can also negatively affect the sensory attributes of products^25–27^. Therefore, detailed profiling of phenolic compounds and their association with sensory attributes of products made from large set of diverse wheat cultivars could be studied.

Fumaric acid varied significantly with respect to release year and showed an increasing trend at a rate of 0.4 mg/kg/year (Table 2, Fig. 2). During dough mixing, the interaction of fumaric acid with gluten proteins was found to reduce the mixing time resulting in rapid dough breakdown and ultimately low loaf volume of bread^28,29^. In the future it would be interesting to compare mixing properties of whole wheat flour from historical and modern cultivars.

Overall, for those metabolites with significant trends over time, the relationship appeared to be increasing non-linearly (Fig. 2). This is similar to what was observed for yield, where a gradual increase was observed with the year of introduction of the cultivars with the landraces similar to the earliest cultivars. Additionally, the heritability of amino acids was lower (*H*^2^ < 0.5) (except isoleucine, tryptophan, and valine) compared to phenolic and fumaric acids (*H*^2^ > 0.5) (Table 2), indicating that these traits were more stable across the harvest years^4,30^.

### Multivariate analysis

**Figure 3:**
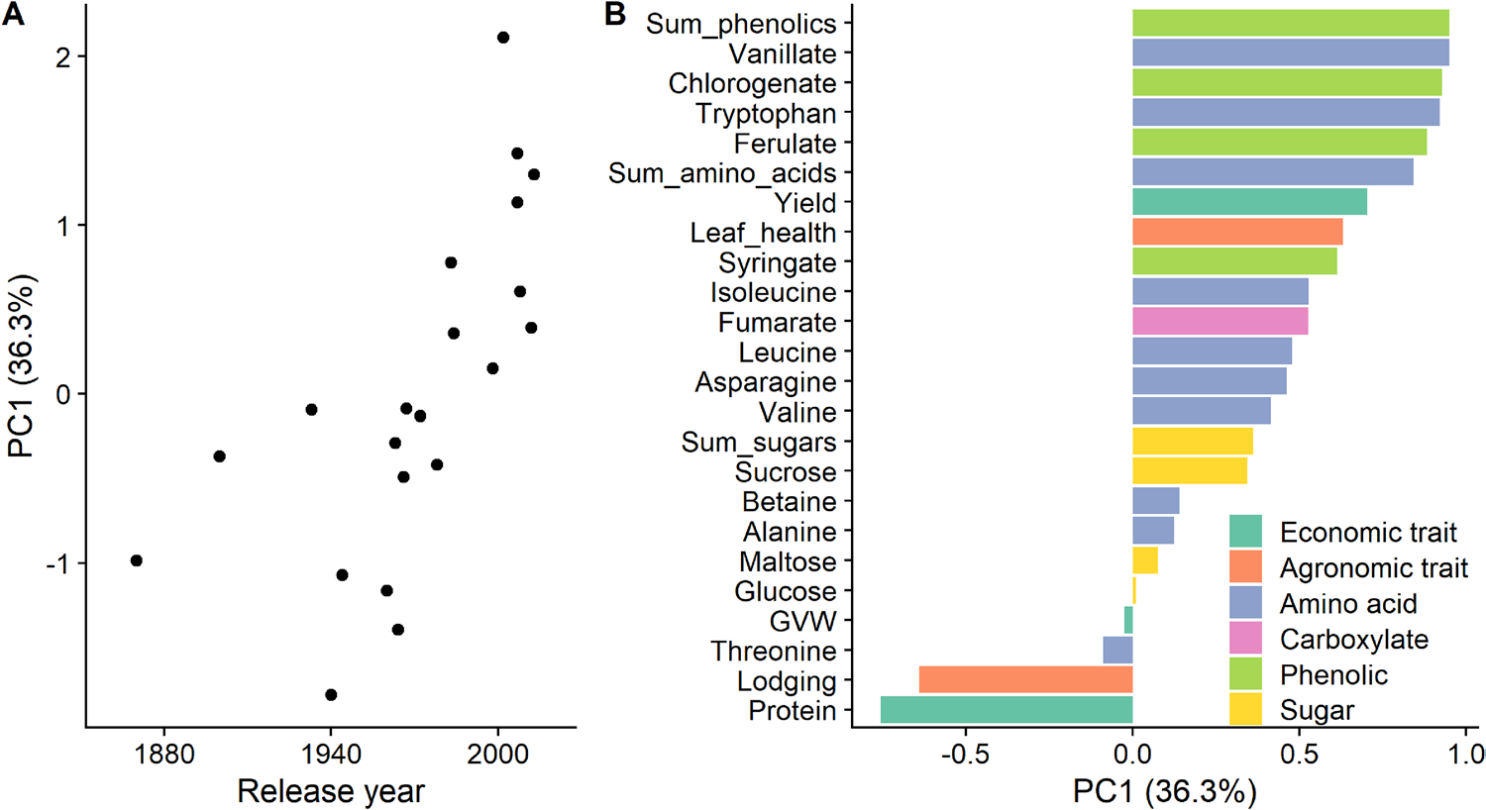
Relationship between cultivar release year and loading on principal component (PC) 1 (**A**) and Eigenvectors of measured variables on PC1 (**B**); plot generated using the ‘ggplot2’ and ‘cowplot’ packages in R^38,40,41^.

A PC analysis was performed on all measured variables to reduce the dimensionality of the data. PC1 accounted for more than one-third of the variance (36.3%) and was the only PC that was significantly correlated with release year (r = 0.68; p < 0.001). Therefore, only PC1 was used for further analysis. As observed for the individual economic and agronomic traits and metabolite data, the scatter plot of release year with PC1 showed that the two landraces (Turkey and Kharkof) were separated from the other cultivars due to their early release years (Fig. 3A). Accordingly, the correlation between PC1 and release year was improved (r = 0.79; p < 0.001) by removing the landraces from the analysis. As evident from Fig. 3A, the modern cultivars were correlated with positive PC1 Eigenvectors while historical cultivars were correlated with negative PC1 Eigenvectors. In Fig. 3B, positive Eigenvectors were observed for many of the amino acids and phenolic compounds (as well as yield and leaf health). Only grain protein and lodging had high negative Eigenvectors on PC1 and were associated with the landraces and historical cultivars.

**Figure 4:**
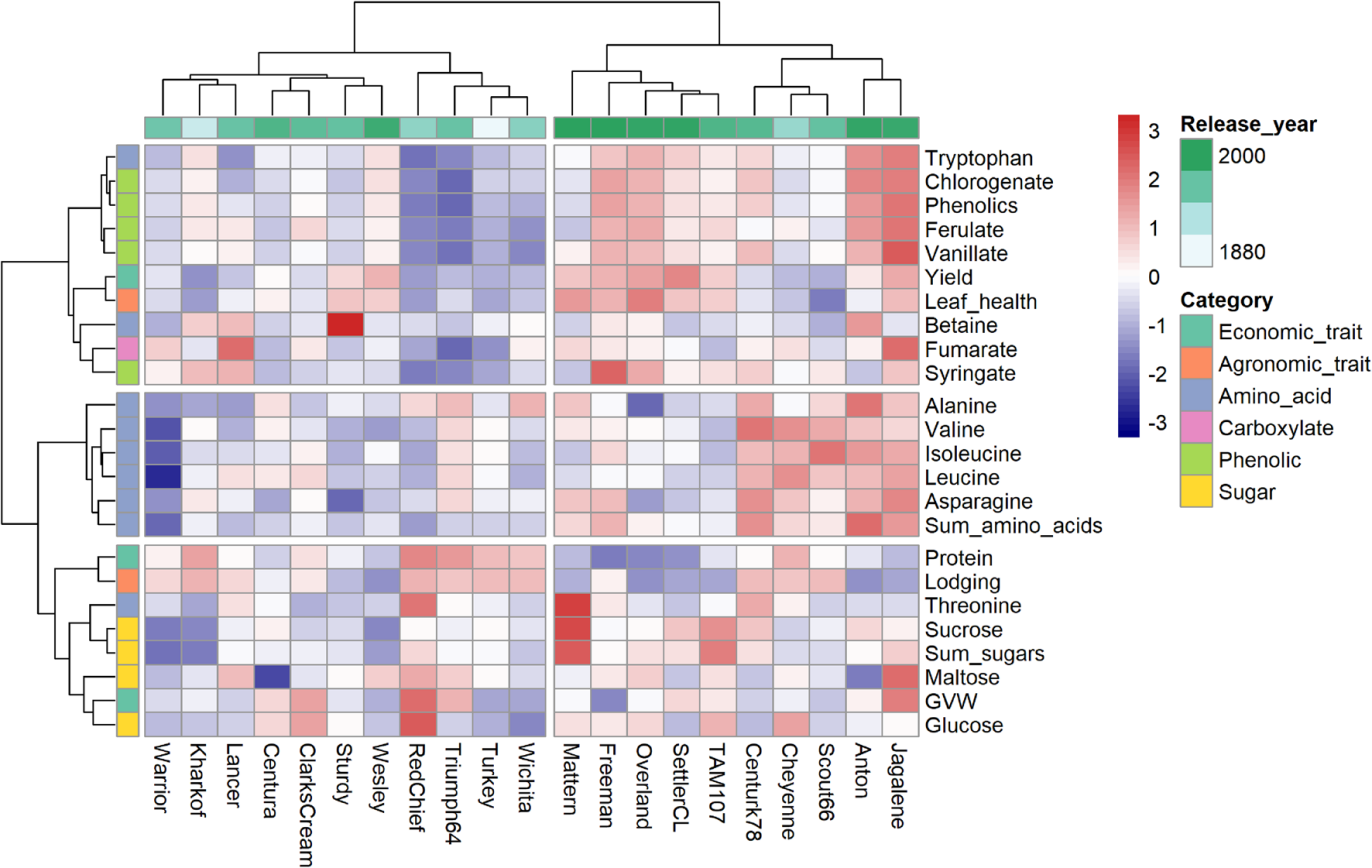
Heat map and hierarchical clustering analysis by Euclidian distance using Ward’s method generated from economic and agronomic traits and wheat metabolites; plot generated using the ‘pheatmap’ package in R^37,38^.

A hierarchical cluster analysis using all economic, agronomic, and wheat metabolite traits revealed two main clusters (Fig. 4). One cluster contained mostly modern cultivars with high concentrations of amino acids and phenolic compounds, while the other cluster contained the landraces and mostly historical cultivars with lower concentrations of these compounds. This is similar to the results from the PC analysis.

The hierarchical cluster analysis also highlighted groupings within the measured variables. Three major groupings were observed. One group consisted only of amino acids, indicating that many of the amino acids are associated with each other. However, another cluster included betaine and tryptophan together with all of the phenolic compounds, suggesting associations between these specific amino acids and phenolic compounds. The last cluster contained the sugars together with protein and lodging.

The results from the PC analysis and hierarchical clustering highlight the changes that have occurred in wheat cultivars across release years. These differences may be due to changes in breeding objectives or due to changes in methodologies used by early and modern breeding programs^31^. The non-linear increase in metabolites with respect to release year was unanticipated, but is in agreement with the investment in wheat breeding programs throughout this time period.

In conclusion, small increases in free amino acid and phenolic acid concentrations were detected in modern cultivars. Multivariate analyses revealed a separation between historical and modern wheats based on economic and agronomic traits and metabolic composition. These differences were attributed to evolving wheat breeding methods, techniques, and objectives, which collectively impacted the composition of wheat. Future studies involving large numbers of diverse genotypes evolved over several decades of breeding may provide additional insights into the compositional, nutraceutical, and functional properties of wheat cultivars.

## Materials and methods

### Plant materials and experimental design

**Figure 5:**
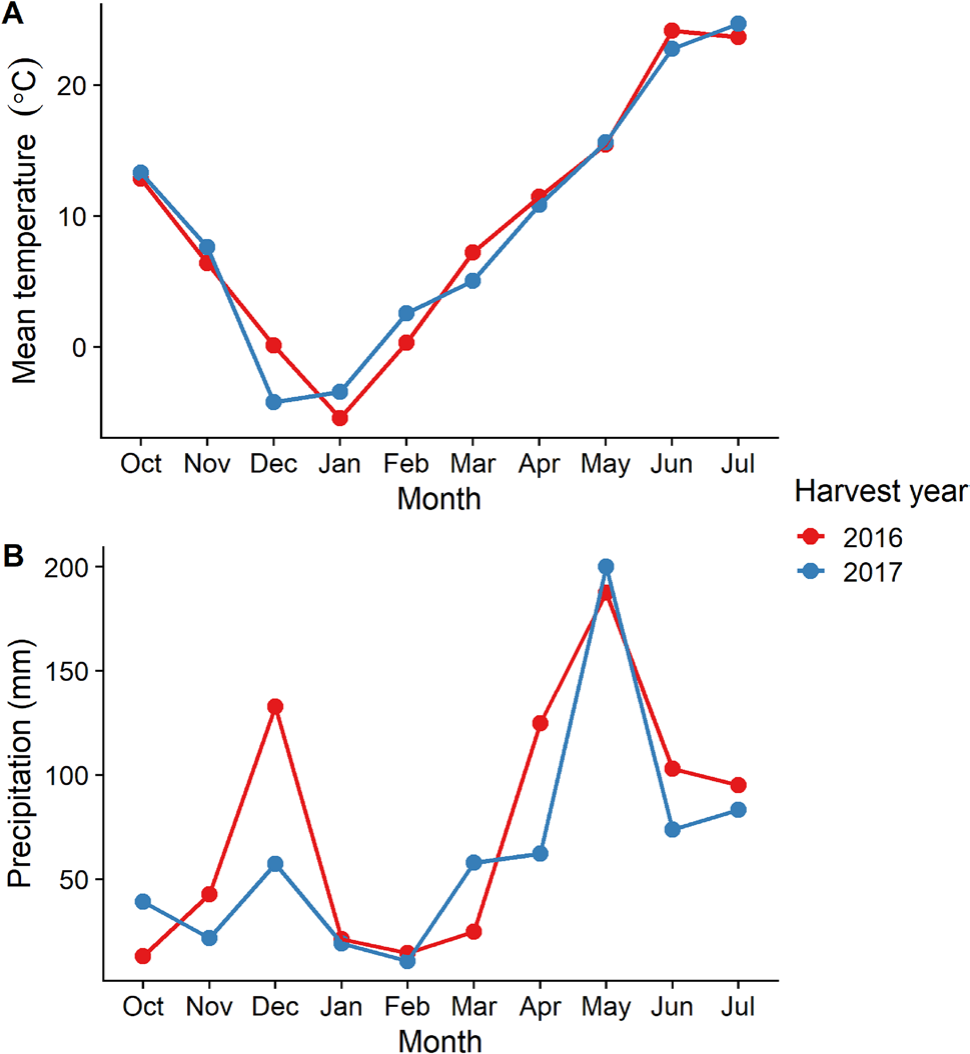
Monthly mean temperature (**A**) and total precipitation (**B**) during the 2016 (2015–2016) and 2017 (2016–2017) winter wheat harvest years at the Eastern Nebraska Research and Extension Center (ENREC) near Mead, NE, USA. Data collected from the Automated Weather Data Network of the High Plains Regional Climate Center^32^; plot generated using the ‘ggplot2’ and ‘cowplot’ packages in R^38,40,41^.

A field experiment was conducted during two harvest years (2016 and 2017) at the agronomy research farm located at Mead, Nebraska, USA (41°13′20.40″ N–96°29′10.79″ W) under rainfed conditions. The experimental design was a randomized complete block design with three replicates. A total of 19 wheat cultivars (*Triticum aestivum* L.) and two landraces were included in the study (Table 1). A landrace was defined as a wheat variety that was not introduced as a result of targeted breeding efforts, while the cultivars were released from formal breeding programs. As the landraces did not have formal release dates, their release years were the approximate year they started to be grown in the region. The 1 m^2^ plots were prepared by applying a nitrogen fertilizer (90 kg/ha) prior to planting in October of the year preceding the harvest year. In the 2016 harvest year, two of the cultivars were planted in less than three replications due to limited seed availability (Red Chief: one replication and Anton: two replications). All replicates were available in the 2017 harvest year. During the active growing season i.e., April to July, mean air temperature ranged from 11.4 to 23.7 °C in 2016 and 10.8 to 24.7 °C in 2017. Similarly, average rainfall ranged from 95 to 187.5 mm in 2016 and 62.2 to 199.9 mm in 2017. These data were collected from the Automated Weather Data Network of the High Plains Regional Climate Center at location Mead 6S station^32^. The weather conditions between the two harvest years included in this study were remarkably similar (Fig. 5). As historical wheats are generally more susceptible to foliar diseases a broad-spectrum fungicide (Caramba, BASF, NJ, USA) was performed at the booting stage at the dose rate recommended by the manufacturer, as is common agronomic practice of the region^33^, to prevent yield and grain quality loss particularly in the more disease-susceptible landraces and cultivars. Seeds from each plot were harvested using a simple plot combine harvester.

### Agronomic traits

Each plot was visually evaluated for foliar disease severity 2 weeks after the fungicide application. To report leaf health, the flag leaf was scored from 0 to 10, where 0 represented a completely brown leaf and 10 a completely healthy green leaf. Lodging was also scored on a scale of 0–10, where 0 represented no lodging and 10 meant all plants were lodged.

### Economic traits

Grain yield and grain volume weight (GVW) were measured as described previously^15,34^. Grain protein concentration was measured using a near infrared reflectance analyzer (DA7250, Perten Instruments, Springfield, IL, USA)^34^.

### Sample preparation for 1H NMR profiling

Seed samples from each plot were taken in bulk and the clean grain samples (~ 25 g) were milled using a cyclone mill (UDY, Fort Collins, CO, U.S.A.) equipped with 1 mm screen. The milled seed was used to quantify metabolites using one-dimensional (1D) ^1^H NMR. The quantification of metabolites present in whole wheat flour was accomplished as described previously^14^. To 30 mg of flour, 1 mL D_2_O:CH_3_OD (80:20, v/v) was added. After extraction at 90 °C for 10 min, the contents were centrifuged at 5000*g* and 4 °C for 10 min and then the supernatant was transferred to a separate tube and kept at 4 °C for 45 min. After centrifuging the tube for an additional 5 min, 0.4 mL of the supernatant was transferred to a 5 mm NMR tube followed by the addition of 0.06 mL internal TMSP-d4 standard [3-(trimethylsilyl)propionic-2,2,3,3-d4, 0.125 mg/mL in D_2_O:CH_3_OD (80:20, v/v)].

### ^1^H NMR profiling

**Figure 6:**
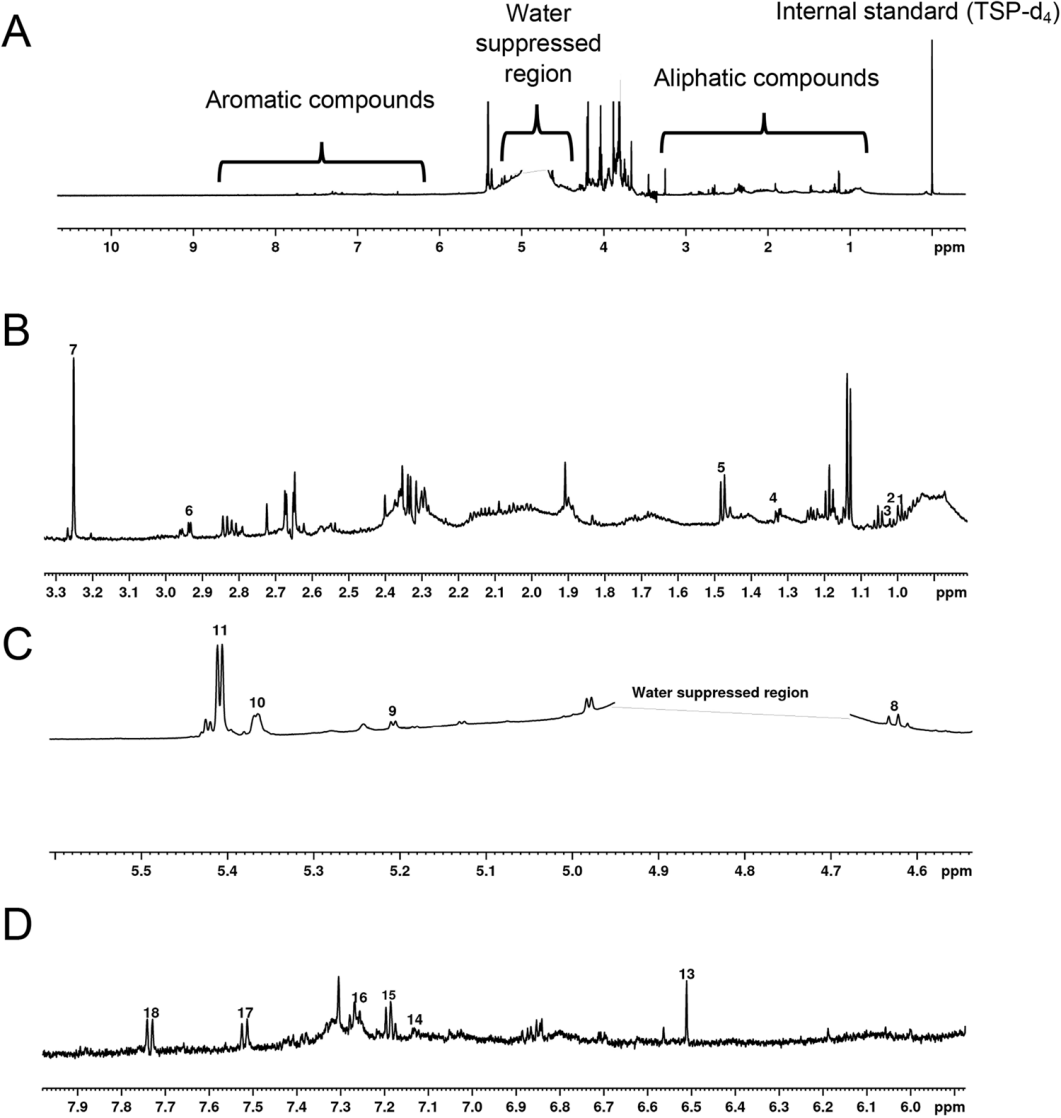
Representative 1D ^1^H NMR spectrum of whole wheat flour (Kharkof) showing full spectrum (**A**), amino acids (**B**), carbohydrates (**C**), and aromatic (**D**) spectral regions. The name of the compounds represented by each number are presented in Table 2.

The 1D ^1^H NMR spectra were acquired using Topspin version 3.5 on a Bruker AVANCE III-HD 700 MHz spectrometer equipped with a 5 mm quadruple resonance QCI-P cryoprobe (^1^ H, ^13^C, ^15^N, and ^31^P) with z-axis gradients using D_2_O:CH_3_OD (80:20, v/v) as the solvent. A SampleJet automated sample changer system, an automatic tune and match system (ATM) and ICON-NMR software was used to automate the NMR data collection. The 1D ^1^H NMR spectra were collected with an excitation sculpting pulse sequence for solvent suppression^35^. The 1D ^1^H NMR spectra were collected at 298 K with a spectral width of 9803 Hz, 64 K data points, 128 scans, 4 dummy scans, and a relaxation delay of 1 s. The NMR spectra were processed using the Chenomx NMR Suite software version 8.4 (Chenomx Inc., Edmonton, Alberta, Canada). The spectra were Fourier transformed, automatically phased and baseline corrected. Chemical shifts and metabolite quantification were referenced to TMSP-d4 peak at δ = 0.00 ppm. The Chenomx library, which consisted of reference spectra for 338 compounds, was used to identify and quantify 16 metabolites from the wheat metabolome extracts. The identified metabolites correspond to four different chemical classes: 1) amino acids (alanine, asparagine, betaine, isoleucine, leucine, threonine, tryptophan, and valine), 2) carbohydrates (glucose, maltose, and sucrose), 3) phenolic acids (ferulic acid, chlorogenic acid, syringic acid, and vanillic acid), and 4) carboxylic acid (fumaric acid). The spectral regions and NMR peak assignments for the identified compounds are highlighted in Fig. 6 and listed in Table 3.

**Table 3:** Metabolites identified in modern and old wheat varieties from 1D ^1^H NMR.

Number on spectrum	Compound	Proton chemical shift (ppm) and multiplicity	Number of protons (H)	Coupling constant (Hz)	Position on compound
1	Leucine	δ 0.97 (t)	6	5.47	8,9
2	Isoleucine	δ 0.98 (d)	3	7.36	9
3	Valine	δ 1.04 (d)	3	7.42	7
4	Threonine	δ 1.33 (d)	3	7.21	8
5	Alanine	δ 1.47 (d)	3	7.56	6
6	Asparagine	δ 2.96 (m)	2	16.52	6
7	Betaine	δ 3.25 (s)	9	–	5,7,8
8,9	Glucose	δ 4.62 (d), 5.20 (d)	1	7.46, 3.60	2
10	Maltose	δ 5.36 (d)	1	3.57	2
11	Sucrose	δ 5.41 (d)	1	3.78	7
13	Fumaric acid	δ 6.5 (s)	2	–	4,5
14	Syringic acid	δ 7.13 (s)	2	–	3,5
15	Chlorogenic acid	δ 7.19 (d)	1	8.51	18
16	Ferulic acid	δ 7.31 (d)	1	7.83	11
17	Vanillic acid	δ 7.51 (d)	1	9.19	6
18	Tryptophan	δ 7.73 (d)	1	8.85	7

### Statistical analysis and graphics

A two-way analysis of variance (ANOVA) was performed using the following model in SAS software (version 9.4, SAS Institute, Cary, NC USA)^36^:

$${y}_{ijkl}=\upmu +{GS}_{i}+ r{(GS)}_{ij}+{RY}_{k }{+GS X RY}_{ik}+{e}_{ijkl}$$

where ${y}_{ijkl}$ is the response variable; µ is overall mean; ${GS}_{i}$ is the effect of ith harvest year (GS); $r{(GS)}_{ij}$ is the effect of jth replication within the ith GS; ${RY}_{k}$ is the effect of kth cultivar year of introduction (release year) which was modeled as a continuous variable; ${GS X RY}_{ik}$ is the interaction effect of ith GS and kth RY; ${e}_{ijkl}$ is the residual. A p < 0.05 was considered statistically significant. Where the effect of release year was significant, least squares means of cultivars were calculated from a separate ANOVA, in which cultivar replaced the RY term in the model and was a fixed variable. These least squares means were used for calculating heritability, plotting the data (release year vs. response variable), principal components (PC) analysis, and for hierarchical cluster analysis using Ward’s minimum variance. PC analysis was performed using SAS software^36^ and cluster analysis was performed using the ‘pheatmap’ package in R (version 4.0.2)^37,38^. In the PC analysis, release year was highly correlated with PC1 and only PC1. Therefore, only PC1 was retained for further analysis rather than showing a typical PC biplot. Broad-sense heritability (*H*^2^) was calculated on a cultivar mean basis in Meta-R (version 6.04)^39^ software using the following equation:

$${H}^{2}=\frac{{\sigma }_{g}^{2}}{{\sigma }_{g}^{2}+\frac{{\sigma }_{gxy}^{2}}{n}+\frac{{\sigma }_{e}^{2}}{nr}}$$

where, *σ*^2^_*g*,_ and *σ*^2^_*gxy*,_ and *σ*^2^_*e*_ were the variance components for cultivars, cultivars × harvest year, and error, respectively, while *n* and *r* were the number of harvest years and replications, respectively.

Figures were generated using ‘ggplot2′, ‘cowplot’, and ‘pheatmap’ packages in R^37,38,40,41^.

## Data Availability

Raw NMR spectra and metadata are available on the Figshare website under (10.6084/m9.figshare.12283880).
